# Do Engineered Nanomaterials Affect Immune Responses by Interacting With Gut Microbiota?

**DOI:** 10.3389/fimmu.2021.684605

**Published:** 2021-09-14

**Authors:** Mingxing Tang, Shuo Li, Lan Wei, Zhaohua Hou, Jing Qu, Liang Li

**Affiliations:** ^1^Huazhong University of Science and Technology Union Shenzhen Hospital, Shenzhen, China; ^2^Institute of Biomedicine and Biotechnology, Shenzhen Institutes of Advanced Technology, Chinese Academy of Sciences, Shenzhen, China; ^3^The 6th Affiliated Hospital of Shenzhen University Health Science Center, Shenzhen, China; ^4^School of Biomedical Science and Pharmacy, Faculty of Health and Medicine, Hunter Medical Research Institute, University of Newcastle, New Lambton Heights, NSW, Australia; ^5^Department of Surgery, Sloan Kettering Institute Z427-D, Mortimer B. Zuckerman Research Center, Memorial Sloan Kettering Cancer Center, New York, NY, United States

**Keywords:** engineered nanomaterials (ENMs), gut microbiota, intestinal permeability, immunomodulation, bacterial components

## Abstract

Engineered nanomaterials (ENMs) have been widely exploited in several industrial domains as well as our daily life, raising concern over their potential adverse effects. While in general ENMs do not seem to have detrimental effects on immunity or induce severe inflammation, their indirect effects on immunity are less known. In particular, since the gut microbiota has been tightly associated with human health and immunity, it is possible that ingested ENMs could affect intestinal immunity indirectly by modulating the microbial community composition and functions. In this perspective, we provide a few pieces of evidence and discuss a possible link connecting ENM exposure, gut microbiota and host immune response. Some experimental works suggest that excessive exposure to ENMs could reshape the gut microbiota, thereby modulating the epithelium integrity and the inflammatory state in the intestine. Within such microenvironment, numerous microbiota-derived components, including but not limited to SCFAs and LPS, may serve as important effectors responsible of the ENM effect on intestinal immunity. Therefore, the gut microbiota is implicated as a crucial regulator of the intestinal immunity upon ENM exposure. This calls for including gut microbiota analysis within future work to assess ENM biocompatibility and immunosafety. This also calls for refinement of future studies that should be designed more elaborately and realistically to mimic the human exposure situation.

## Introduction

Unique properties including large surface area, high catalytic properties and antimicrobial efficacy confer to engineered nanomaterials (ENMs) a significant range of applications in nanomedicine and consumer products (1, 2), raising public concerns about their biosafety. For example, nanoparticulate Ag, TiO_2_, ZnO and plastics are widely used in food additives (3), components of food packaging and containers (4, 5), and toothpaste (6). Oral exposure to these ENMs in our daily life is therefore likely through ingestion of food or water that deliberately or inadvertently contain ENMs. ENMs might therefore reach the gastro-intestinal tract (GIT) and interact with mucosal cells. Indeed, endocytosis of ENMs by intestinal epithelial cells (IECs) and various immune cells is observed using either conventional 2D *in vitro* models such as tumor cell lines (7, 8) or *in vivo* animal models (9). Moreover, it has been reported that ENMs could modulate innate/inflammatory immune responses upon direct interactions with neutrophils, macrophages, dendritic cells (DCs) and the complement system (10–13). Upon ingestion, ENMs most likely also come in contact with gut microbiota, *i.e.*, the population of microbes residing in the intestinal lumen and mucosa. It has been long known that the gut microbiota is essential for the development of the immune system and for immune homeostasis (14). Recent observations suggest that the ENM effects on innate/inflammatory responses largely depend on the co-presence of bacterial agents such as lipopolysaccharide (LPS) (15, 16). Thus, it is a logical assumption that ENMs could affect immunity by altering gut microbiota, a concept that is currently unexplored.

Herein, we provide an overview of the current state-of-the-art, and discuss a hypothetical scenario in which ingested EMNs may affect host immunity by modulating the gut microbiota. From published *in vivo* studies in different models and with different ENMs, a high level of variability is found regarding the ENM effects on gut microbiota and local/systemic immunity (**Table 1**).

**Table 1:** Representative *in vivo* assays studying the impact of ENMs on gut microbiota and subsequent influences on intestinal immunity.

Engineered NanoMaterials	Animal model	Exposure dose	Exposure way and duration	Analysis methods of gut microbiota	Gut microbiota changes by ENM treatment	Immune markers	Clinical effect/Immune response	References
Silver nanoparticles with a diameter of 55 ± 3 nm	3 mo-old C57BL/6 female mice	0, 11.4, 114 and 1140 μg/kg bw/dy	Dietary exposure for 28 days	16S rRNA Sequencing of Bacterial DNA from Fecal Samples	*Odoribacteraceae, Bacteroidaceae* and S24-7 family decreased while *Lactobacillaceae* and *Lachnospiraceae* increased	Serum C-reactive protein level; histology of ileum villi, intestinal goblet cells, glycocalyx and colon	No overt effect on body weight gain, the intestinal histology as well as the serum C-reactive protein level.	(17)
Silver nanoparticles with a diameter of 12 ± 3 nm	7 wk-old CD-1 (ICR) male mice	2.5 mg/kg bw/dy	Oral gavage daily for 7 days	Pyrosequencing of 16S rRNA genes in fecal samples	*Firmicutes/Bacteroidetes* ratio reduced. *Alistipes*, *Bacteroides* and *Prevotella* increased, while *Lactobacillus* decreased	Blood cell level, serum lymphocyte level. colon length, disease activity index (DAI), histology of colon; intestinal permeability; IL-1β, IL-6 and TNF-α in small bowel and colon	The level of blood cells and lymphocytes was increased; Body weight decreased and colon length was shortened by Ag NP; The epithelial architecture and crypts in colon was destroyed. Intestinal permeability was significantly increased; Pro-inflammatory cytokines: IL-1β, IL-6 and TNF-α were upregulated.	(18)
Silver nanoparticle with a diameter of 294 nm	6 wk-old BALB/c male mice	5 ng/dy	Oral gavage daily for 4 days	A few specific bacteria from the colon mucosa were isolated and counted by selective plates	*Lactobacillus* sp. decreased, while *Clostridium perfringens* and *Escherichia coli increased but not significantly*	Stool consistence; colon length and weight; colon epithelial histology; myeloperoxidase activity in the colon. Colon smooth muscle thickness; Presence of ulcers, hemorrhage, fecal blood, and diarrhea.	NanoAg1 displayed weaker anti-inflammatory effect and alleviated the TNBS-induced severe colonic injury.	(19)
Silver nanoparticle with a diameter of 122 nm					*Lactobacillus* sp. increased while *Clostridium perfringens and Escherichia coli* decreased		NanoAg2 significantly attenuated DSS-induced colitis and alleviated the TNBS-induced severe colonic injury.	
PVP-stabilized silver nanoparticulate with a diameter of 14 nm	4 wk-old Wistar Hannover Galas rats	2.25, 4.5 or 9 mg/kg bw/dy	Oral gavage daily for 14 days and 28 days	Bacterial phyla in caecum content were quantified by qPCR	No significant change	Histology of liver, kidney, ileum and myocardium. Twenty-four-hour urine and feces.	No overt effect on body weight gain, organ weight, organ histology and leucocyte infiltration	(20)
PVP- or citrate-coated silver nanoparticles with a diameter of 20 and 110 nm	10-12 wk-old C57BL/6NCrl male mice	10 mg/kg bw/dy	Oral gavage daily for 28 days	16S rRNA sequencing of contents in the cecal tips	No significant change	Not studied	Not studied	(21)
TiO_2_ nanoparticles with a diameter of 17 ± 2 nm	7 wk-old CD-1 (ICR) male mice	2.5 mg/kg bw/dy	Oral gavage daily for 7 days	Pyrosequencing of 16S rRNA genes in fecal samples	*Bacteroides* decreased	Blood cell level, serum lymphocyte level. colon length, histology of colon; intestinal permeability; IL-1β, IL-6 and TNF-α in small bowel and colon	TiO_2_ ENMs were deposited in the stomach and the colon; no effect on body weight, no significant change in DAI index and colon length, loss and shortening of crypts, inflammatory cell infiltration and mucosal erosions but a few inflammatory cells scattered within duodenal and colonic sections; The integrity of the GIT epithelium is intact; IL-1β level was increased in the small bowel and colon.	(18)
Spherical anatase TiO_2_ nanoparticles with a diameter of 20 nm in water, of 134 ± 22 nm in gastric fluid, of 420 ± 25 nm in intestinal fluid	8 wk-old C57BL/6 male mice	100 mg/kg bw/dy	Oral gavage daily for 28 days	16S rRNA Sequencing of Bacterial DNA from Fecal Samples	*Bacteroides* and *Akkermansia* increased	Histology of liver, spleen, kidney, lung, heart, brain, jejunum and colon. NP deposition in these organs mentioned.	No effect on body weight or histology of key organs	(22)
Edged conner rutile TiO_2_ nanoparticles with a diameter of 16 nm in water, of 148± 30 in gastric fluid, of 361 ± 8 nm in intestinal fluid					*Escherichia-Shigella and Rhodococcus* increased, while *Bacteroidetes* and *Firmicutes* decreased		Intestinal villi length increased and villus epithelium cells became irregularly arranged	
Spherical anatase TiO_2_ nanoparticles with a diameter 29 ± 9 nm	3 wk-old Sprague-Dawley rats	0, 2, 10, 50 mg/kg bw	Oral gavage daily for 30 days	16S rRNA Sequencing of Bacterial DNA from Fecal Samples	Increased abundance of *L. gasseri*, *Turicibacter*, and *L.* NK4A136_group and decreased abundance of *Veillonella*	Body weight; LPS and short-chain fatty acids content in the feces; colon histology; fecal metabolites; presence of glutathione, glutathione peroxidase, lipid peroxidation products, superoxide dismutase, and sulfhydryl groups in tissue homogenates; Inflammatory cytokines in serum	Accumulation of malondialdehyde and decreased activity of superoxide dismutase were detected in colon tissues; Increased concentration of IL-6 in the serum. The number of goblet cells decreased and inflammatory cells infiltrated in colon epithelium.	(23)
ZnO nanoparticles with a diameter of average 71.61 nm	28 dy-old weaned piglets	150, 300, or 450 mg/kg in diet	Dietary exposure for 21 days	The cecal, colonic and rectal contents were spread on selective plates to assess *E. coli, Salmonella, Lactobacillus, and Bacillus bifidus*	*E. coli* decreased	Histology of the jejunum, duodenum and ileum; serum cytokines and immunoglobins	Significant improvements in average daily weight gain, average daily feed intake and gain to feed ratio were observed. The diarrhea rate was reduced. The villus height in the jejunum, duodenum and ileum was increased. The blood concentration of IgA, serum concentrations of IL-6 and TNF-α was increased; while the blood concentration of IgM was decreased.	(24)
ZnO nanoparticles with a diameter of 23-25 nm	27 dy-old weaned piglets	600 mg/kg in diet	Dietary exposure for 14 days	16S rRNA sequencing of the intestinal contents	*Lactobacillus* increased while *Prevotella* and *Oscillospira* decreased in the colon	Histology of jejunal tissue; gene expression of pro-inflammatory cytokines, cell proliferation markers, antioxidant markers, tight junction proteins and cell death markers in the jejunal tissue	The diarrhea incidence was reduced; average daily gain and feed intake were unaltered; villus height as well as the ratio of villus height to crypt depth was increased; the expression of antioxidant enzymes and tight junction in the jejunal tissues was increased significantly; the expression of cell proliferation markers was increased; the expression of pro-inflammatory markers was reduced.	(25)
SWCNT with a diameter of 1nm and a length of 1-5 μm	7 wk-old CD-1 (ICR) male mice	0.05, 0.5, and 2.5 mg kg/bw/dy	Oral gavage daily for 7 days	16S rRNA sequencing of fecal samples	*Bacteroides, Prevotella*, and *Alistipes* increased, while *Bacteroidales, Lachnospriaceae* and *Lactobacillus* decreased	Intestine histology, intestinal epithelium permeability, cytokine production in both duodenum and colon and lymphocyte abundance in the serum.	Ulceration, crypt damage, and inflammatory cell infiltration were observed in the duodenum and colon. The intestinal permeability was significantly increased. IL-1β, IL-6, and TNF-α increased in the duodenum and the colon. White blood cell, lymphocytes, and intermediate cell counts significantly elevated in the serum.	(26)
MWCNT with a diameter of 8 ± 1 nm and a length of 0.5-2 μm		2.5 mg kg/bw/day		16S rRNA sequencing of fecal samples	*Bacteroides, Prevotella, Alistipes*, and *Ruminococcaceae* increased, whereas *Bacteroidales, Lachnospriaceae* and *Lactobacillus* decreased		Slight microvilli damage and inflammatory cell infiltration in duodenum and a few inflammatory cell infiltrations in colon. Significant increase of intestinal permeability and the elevated levels of proinflammatory cytokines IL-1β, IL-6, and TNF-α in duodenum and colon were observed.	
Graphene oxide nanoparticles with a thickness of 1-2 μm and a dimension area of 1-14 μm^2^		2.5 mg kg/bw/dy		16S rRNA sequencing of fecal samples	*Lachnospriaceae, Lactobacillus, Ruminococcus, Alistipes, Oscillibactyer*, and *Prevotella* increased; while *Bacteroidales* and *Bacteroides* decreased		Slightly pathological changes of epithelium loss and inflammatory cell infiltration in duodenum. Significant increase of intestinal permeability and the elevated levels of proinflammatory cytokines IL-1β, IL-6, and TNF-α in duodenum and colon were observed.	
Lysine-modified SWCNT with a length of 400 nm and a diameter of 2-3 nm	23-30 dy-old BALB/c mice	4.25 mg/wk	Oral gavage or intraperitoneal dosing weekly for 7 or 8 weeks	16S rRNA sequencing of fecal samples	The α- and β-diversity of the mouse microbiota reduced in the cecum but not in colon or ileum.	Body weight, liver and kidney weight.	No overt effect on body weight as well as liver and kidney weights	(27)
Polyethylene microplastics with a diameter of 10-150 μm	C57BL/6 mice	6, 60, and 600 μg/dy	Dietary exposure for 5 weeks	16S rRNA sequencing of fecal samples	The α- and β-diversity of the mouse microbiota increased. Staphylococcus increased, while Parabacteroides decreased	Serum cytokine; T cells in the spleen; TLR4, AP-1, and IRF5 expression; intestinal histology.	Serum concentrations of IL-1α increased; the percentage of Th17 and T_regs_ cells among CD4^+^ cells decreased; edema occurred and lymphocyte and plasma cell infiltration was observed in the lamina propria of the colon and duodenum; TLR4, AP-1, and IRF5 expression significantly increased in the colon and duodenum.	(28)
Cuboid CuO nanoparticles with a dimension area of 20 nm by 50 nm	*Eisenia fetida* with a weight range between 300 and 600 mg	160 mg/kg soil	Exposure to soil containing ENMs for 28 days	16S rRNA sequencing of microbiota in gut tissue	*Candidatus Lumbricincola* and *Luteolibacter* decreased	Histology of the gut epithelium and longitudinal muscle tissue; expression of coelomic cytolytic factor, lysenin/fetidin and lysozyme.	No overt effect on tissue integrity, and immune responses	(29)

Doses relevant for human exposure level are marked using underline. AP-1, activating protein-1; Bw, body weight; CuO, copper oxide; DSS, dextran sulfate sodium; Dy, day; GIT, gastrointestinal tract; IL, interleukin; Ig, immunoglobin; IRF5, interferon regulatory factor 5; LPS, lipopolysaccharide; Mo, month; MWCNT, multiple-walled carbon nanotubes; PVP, polyvinyl pyrrolidone; SWCNT, single-walled carbon nanotubes; Th17, T helper type 17; TNBS, trinitrobenzene sulfonic acid; TLR4, Toll-like receptor 4; TNF, tumour necrosis factor; Wk, week; ZnO, zinc oxide.

## LPS and SCFAs: Two Representative Microbial Molecules Bridging Gut Microbiota and Intestinal Immunity

**Figure 1:**
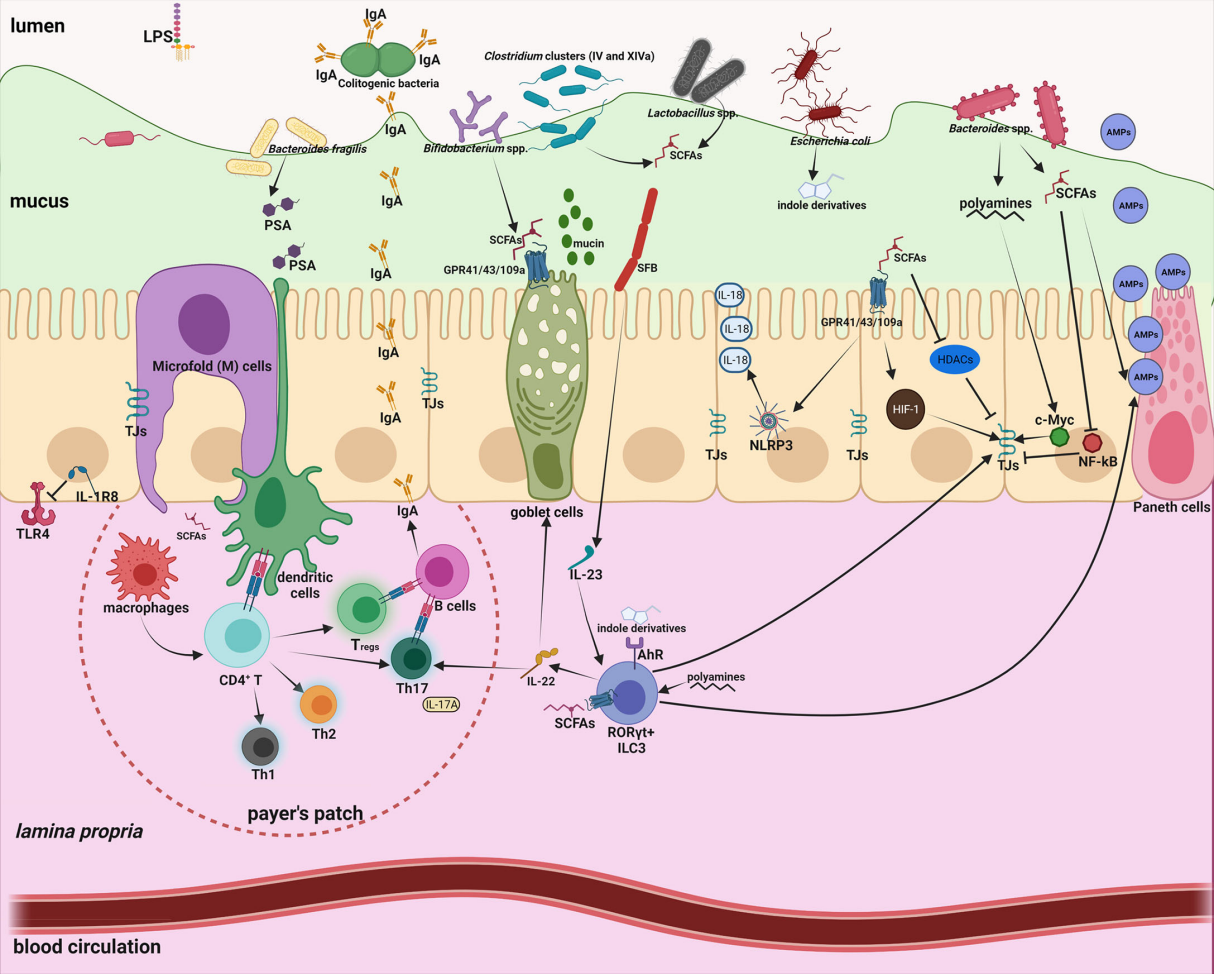
Intestinal homeostasis is tightly controlled by gut microbiota through a large number of microbial metabolites/components. Intestinal mucus not only provides a habitat for bacterial colonization but also serves as a lubricant barrier to restrict most gut microbes in the outer layer. Microfold (M) cells above the Peyer’s patch are essential to transport microbiota-derived metabolites/components to maintain the homeostasis of the mucosal immune system. 1) The effects of short-chain-fatty-acids (SCFAs) are manifold, including enhanced mucus production; inhibition of nuclear factor-κB (NF-κB); activation of NLR-family-pyrin-domain-containing-3 (NLRP3) inflammasomes and subsequent production of interleukin-18 (IL-18); enhanced antimicrobial peptide (AMP) production; polarization of anti-inflammatory macrophages; increased Immunoglobulin A (IgA) secretion; reduced expression of T cell-activating molecules on antigen-presenting cells; and increased number and function of colonic regulatory T (T_regs_) cells. 2) Polyamines can activate ROR^γt+^ group 3 innate lymphoid cells (ILC3) and induce production of IL-22, which promote mucus and AMP secretion, and ensure commensal compartmentalization from the intestinal epithelium. 3) Indole derivatives produced by gut commensals can stimulate Aryl-Hydrocarbon-Receptor (AhR) to activate ILC3 and fortify the epithelium barrier function. 4) Polysaccharide A (PSA) from *Bacteroides fragilis* is taken up by DCs, processed and presented to naive CD4^+^ T cells, inducing the expansion of FOXP3^+^ T_reg_ cells. 5) Attachment of segmented filamentous bacteria (SFB) to the epithelium enhances differentiation and expansion of CD4^+^ Th17 cells. Foxp3^+^ Treg cells and Th17 cells localize in the Peyer’s patches, and induce B cell class-switch and IgA production, which in turn remodels microbiota. 6) Basolateral location of the LPS receptor TLR4 on IECs and expression of the anti-inflammatory IL-1R8 allow proper immune tolerance.

Mounting evidence has highlighted the tremendous contribution of gut microbiota to human physiology (30–35). Within this microbiota-immune system interaction, a large amount of microbial metabolites and components serve as potent effectors to orchestrate their communication (36, 37). We will specifically discuss hereafter the immunomodulatory effects of short-chain fatty acids (SCFAs) and LPS. More comprehensive information is shown in **Figure 1** and extensively discussed in other excellent reviews (32, 33, 36–39). SCFAs are generated from indigestible oligosaccharides by gut commensals, including *Lactobacillus*, *Bacteroides*, *Bifidobacterium*, *Feacalibacterium*, *etc.* (40). LPS is the major membrane component of Gram-negative bacteria and has profound immunostimulatory and inflammatory capacity (41). The immunological effects of these microbiota-derived molecules are manifold, covering innate and adaptive immunity.

### Regulation of Innate Immunity

As a physical barrier at the intestinal surface, IECs are equipped with an array of immune receptors to sense and integrate microbiota-derived metabolites and components for maintaining immune homeostasis. By activating G-protein-coupled-receptors (GPR41, GPR43, GPR109A) on IECs, SCFAs can promote the activation of the NOD-like-receptor-protein 3 (NLRP3) inflammasome, inducing production of the homeostatic cytokine interleukin-18 (IL-18) (42). SCFAs can also stimulate goblet cell differentiation, mucin gene transcription and mucus secretion (43). Pattern recognition receptors (PRRs) on the IEC surface, such as Toll-like receptors (TLRs), can sense microbial antigens. Notably, a number of homeostatic mechanisms ensure immune tolerance towards commensals, such as the basolateral location of the LPS receptor TLR4 that allows binding and activation only to invading bacteria (44) and the constitutive expression of the anti-inflammatory IL-1R8, which binds to and inhibits TLR and IL-1 receptors (45).

Intriguingly, the commensal gut microbiota also interacts with IECs to maintain an effective gut barrier. SCFAs, particularly butyrate, have crucial roles in regulating tight junction (TJ) proteins *via* multifaceted signaling pathways (46), such as HIF-1 stabilization (47), and histone deacetylase (HDAC) inhibition (48). By contrast pathogenic *E. coli* Shiga-toxins and LPS (49) could compromise the epithelial barrier by disrupting TJ. LPS increases intestinal epithelium permeability through the TLR4/MyD88/TGF-β activated kinase 1 (TAK1)/nuclear-factor-κB (NF-κB) cascade in both *in vitro* and *in vivo* models (50).

Immunoregulation of gut microbiota also covers innate lymphoid cells (ILCs), a subpopulation of innate cells (natural killer cells, ILC1, ILC2, ILC3) specialized in recognizing and reacting to infectious challenges. SCFAs can modulate ILC3 proliferation and stimulate IL-22 production in an AKT/STAT3-dependent manner. IL-22 promotes antimicrobial peptide (AMP) production, mucin secretion and colonization of commensal microbes (51).

Intestinal resident macrophages maintain the tissue homeostasis by removing senescent and anomalous cells, and contribute to tissue defense by eliminating invading pathogens and foreign objects. Upon binding to TLR4, LPS can promote inflammatory macrophage activation (M1 polarization), with the production of an array of inflammatory cytokines, IL-1β, IL-6, IL-12 and tumor necrosis factor-α (TNF-α) (52). Conversely, SCFA butyrate facilitates the anti-inflammatory/tissue-healing macrophage polarization, probably by activation of the H3K9/STAT6 signaling pathway (53).

### Regulation of Adaptive Immunity

The impact of gut microbiota goes beyond the innate immunity, through its ability to affect the activation of antigen-presenting cells (APCs), which are the link between innate and adaptive immunity. APCs in the gut encompass resident DCs and tissue macrophages, which are involved in antigen presentation to naïve and primed T cells. Activation, maturation and functionality of DCs and macrophages can be influenced by LPS and SCFAs. As the major APCs in the intestine (54), macrophages can be regulated by microbial niacin and butyrate *via* activating GPR109A, which in turn increases production of anti-inflammatory IL-10 and Aldehyde-Dehydrogenase-1-Family-Member-A1 (ALDH1A), and induces differentiation of T cells (55). LPS is a potent elicitor of DC migration and maturation by activating mitogen-activated protein kinase (MAPK) and NF-κB signaling pathways (56). SCFAs can block the DC generation from bone marrow stem cells (57), and down-regulate expression of the T cell-stimulatory proteins CD80, CD83 and major-histocompatibility-complex class II (MHCII) (58).

Through its effects on APCs that produce several cytokines necessary for T cell activation, the gut microbiota is also involved in differentiation of naïve CD4^+^ T cells into defined subsets, including T helper (Th1, Th2 and Th17) and regulatory T cells (T_regs_). Inhibition of HDAC by SCFAs can regulate the mTOR–S6K pathway required for generation of Th17, Th1, and IL-10^+^ T cells (59). T_regs_ have important anti-inflammatory roles, allowing the immune system to tolerate antigens derived from gut microbiota and diet. Through binding to GPR43, SCFAs can stimulate T_regs_ proliferation (60). Additionally, SCFAs control the expression of genes necessary for plasma B cell differentiation and Immunoglobulin A (IgA) production (61). As the largest class of immunoglobulins in the intestinal mucosa, IgA targets microbial antigens and preferentially coats colitogenic bacteria, therefore preventing inflammation and perturbation of intestinal homeostasis (62).

## Numerous ENMs Could Reshape the Gut Microbiota Signature but It Is Not a General Effect

**Figure 2:**
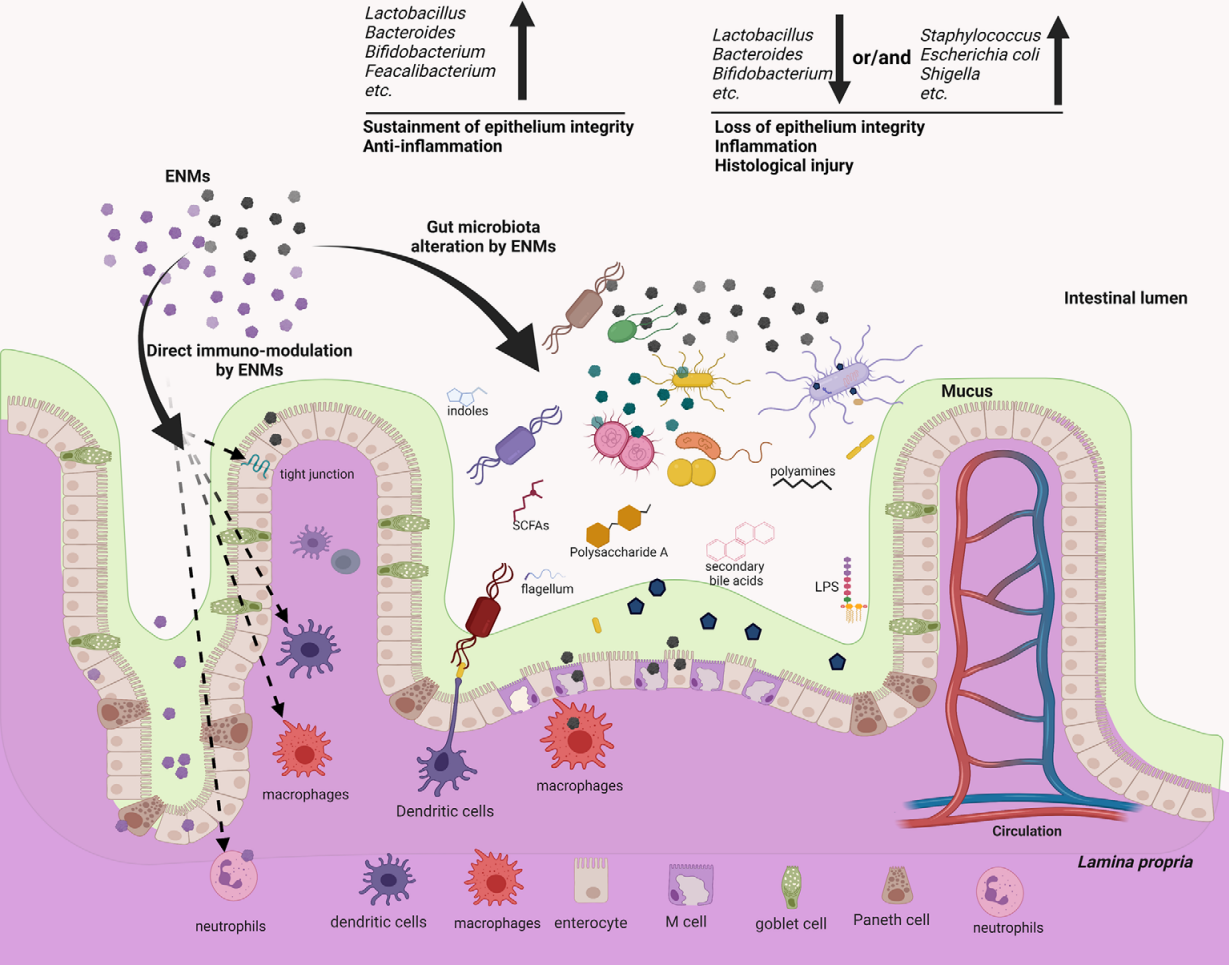
ENMs could not only modulate several components of the mucosal immune systems directly, but also reshape the gut microbiota, which may potentially act as an alternative but important regulator to mediate the immuno-modulatory effects of ENMs. ENMs could accumulate and directly interact with neutrophils, macrophages, dendritic cells (DCs) and the complement system to modulate innate/inflammatory immune responses. On the other hand, several metallic and non-metallic ENMs are proved to be bactericidal, either impairing the bacterial membrane, or causing intracellular oxidative stress, or generating genotoxicity. As responses to the ENM bactericidal effects, members of gut microbiota may rapidly develop resistance, but the associated molecular strategies and efficacy often differ among distinct members. Many *in vitro* and *in vivo* assays showed that ENMs can alter the gut microbiota profile, enrich the relative abundance of pathogens or decrease that of gut commensals. This effect often associates with intestinal inflammation and tissue injury. While some ENMs could increase gut commensals, which in turn exert anti-inflammatory effects. Conversely, a few works show that the gut microbiota remains resilient following oral exposure to ENMs, indicating that the ENM effect on gut microbiota/mucosal immunity is not general.

ENMs might interact with gut microbes in different manners (**Figure 2**). Of special concern is the intrinsic antimicrobial potency of some ENMs. Nanoparticulate Au, Ag, TiO_2_ and ZnO can exert bactericidal activity by disrupting the bacterial membrane (63, 64), inducing intracellular reactive oxygen species (64, 65) and causing direct genotoxicity (66). Conversely, iron oxide and graphene ENMs can promote the growth of some bacterial species, with mechanisms still largely unknown (67, 68). Adding to the complexity is that many gut microbes could rapidly develop strategies to resist ENM bactericidal actions (69). Gram-negative bacteria are thought to be more tolerant to ENMs, in that a lower amount of the negatively charged peptidoglycan may be less effective in trapping the positively-charged metal ions, while other studies argue that Gram-positive bacteria have thicker membranes to ensure stronger protection (69, 70). The resistance mechanism to ENMs could be specific at the bacterial species/strain level. The gut microbiota remodeling effect of ENMs has been substantiated by a panel of *in vivo* assays. For instance, dietary Ag ENMs for mice decreased *Odoribacteraceae, Bacteroidaceae* and the S24-7 family while increasing *Lactobacillaceae* and *Lachnospiraceae* (17). Oral gavage of TiO_2_ ENMs in mice also modulated the gut microbiota, with *Bacteroides *and* Akkermansia* increased (22). Oral administration of non-metallic single-walled carbon nanotubes (SWCNTs) modestly altered the α- and β-diversity of the mouse microbiome (27). The modulation of animal gut microbiota by other ENMs is systemically summarized in recent reviews, which include nanoparticulate plastics, graphene oxide, multi-walled carbon nanotubes (MWCNT), SWCNT, Ag, ZnO, MoO_3_, MoS_2_, TiO_2_, CuO and SiO_2_ (5, 71, 72). Numerous *in vitro* assays also validate the ENM modulatory effect on gut microbiota samples (68, 73–75). Importantly, there appears to be no consensus effect, as multiple ENM-related factors (dose, physicochemical nature, particle size, surface charge, shape and stability) might dictate their modulatory mechanisms and efficacy (64, 76). In addition, the gut microbiota signature varies among individuals, and even within the same subject it changes with time, food intake and health conditions (77–79).

One may suspect the modulatory effect of ENMs on gut microbiota. Because the link between immunity and microbiota is bidirectional (32), could it be possible that ENMs affect immunity and as a consequence the microbiota? Indeed, ENMs could accumulate in the intestine, favor inflammatory responses and impair the barrier function, including IEC apoptosis, tight junction opening, decreased AMP production, Th1/T_regs_ imbalance, aberrant IgA secretion and inflammatory activation of macrophages (72). In this situation, the gut microbiota can be in turn altered by the mucosal immunity. But, a considerable number of ENMs (Ag, SWCNT, CuO, TiO_2_) are shown to alter the gut microbiota without inducing any detectable changes in intestinal immunity (17, 22, 27, 29). These data suggest that ENMs may cooperate with the mucosal immunity to modulate the gut microbiota.

## The Potential Effect Of ENM-Altered Microbiota on Intestinal Inflammation

As discussed above, microbiota-derived metabolites such as SCFAs have important roles in the regulation of gut immunity (**Figure 1**), while ENM exposure that reduces SCFA-producing bacteria may perturb the immune homeostasis and cause inflammation (**Table 1**). Indeed, gut microbiota dysbiosis appears to tightly associate with inflammatory bowel disease (IBD), a chronic and relapsing inflammatory disorder of the intestine (80). This link has been observed in several *in vivo* assays that model GIT exposure to ENMs. Oral administration of Ag ENM (2.5 mg/kg body weight daily) in mice profoundly reduced the *Firmicutes* to *Bacteroidetes* ratio, specifically due to an increase in *Alistipes*, *Bacteroides* and *Prevotella*, and a significant decrease in SCFA-producing *Lactobacillus*. The altered microbiota could cause some IBD-like symptoms, including disrupted epithelium structure, increased intestinal permeability and upregulation of inflammatory cytokines (IL-1β, IL-6 and TNF-α) (18). In the same study, oral gavage of TiO_2_ ENMs (2.5 mg/kg body weight daily) significantly decreased the probiotic *Bacteroides* and triggered a low-grade colonic inflammation (18). Likewise, administration of SWCNT, MWCNT and graphene oxide ENMs (2.5 mg/kg body weight daily) in mice disrupted the gut microbiota signature, with commensal *Lactobacillus* and *Bacteroides* decreased. The exposed mice displayed tissue injury, increased intestinal permeability and elevated production of inflammatory IL-1β, IL-6, and TNF-α (26).

Moreover, enrichment of pathogens and associated virulence factors following ENM administration could also cause intestinal inflammation (81). The work of Chen et al. showed that oral administration of TiO_2_ ENMs (50 mg/kg body weight) in rats decreased the number of goblet cells, elicited immune cell infiltration and mitochondrial abnormalities in the colon tissues, suggesting redox imbalance and inflammation. TiO_2_ ENM treatment remarkably affected the fecal metabolite profile, and particularly enriched the LPS content (23). In another work, oral gavage of TiO_2_ ENM (100 mg/kg body weight daily) in mice impaired the intestinal microvilli structure, and increased *Escherichia* and *Shigella*, two potential pathogens for elicitation of intestinal inflammation (22). Dietary nanoplastics (600 μg daily) for mice significantly increased pathogenic *Staphylococcus* abundance alongside a decrease in *Parabacteroides* (28). The ENM-feeding group displayed a chronic intestinal inflammation, such as increased serum IL-1α, abnormal ratio of Th17 and T_regs_ among CD4^+^ cells, infiltration of lymphocytes and plasma B cells in the *lamina propria*, and higher expression of inflammatory markers (TLR4, AP-1, and IRF5) (28). In these cases, ENMs may enrich opportunistic pathogens or liberate the membrane-bound PAMPs from bacterial cells (82). The inflammatory antigens, such as LPS, exotoxin and flagellin, would bind to PRRs on IECs and immune cells, thus activating inflammatory pathways and promoting an excessive intestinal inflammation (83–86). However, most of these *in vivo* studies based on animal models rarely simulated the realistic human exposure condition. **Table 1** details such shortcomings: either subjects were exposed to an excessive dose of ENMs, or ENMs were administered alone without food which is not a real-life fashion. Whether ENMs were contaminated by LPS was not checked, either.

There is no general effect of ENMs on gut microbiota and intestinal immunity. Contrary to the aforementioned adverse effects, other studies showed that ENM ingestion can increase commensal microbes and exert anti-inflammatory effects. Dietary ZnO ENMs (600 mg/kg food) for weaned piglets increased *Lactobacillus*, leading to upregulation of tight junction proteins and antioxidant enzymes, and decreased expression of inflammatory interferon-γ (IFN-γ), IL-1β, TNF-α and NF-κB (25). Similarly, oral gavage of Ag ENMs (5ng daily) attenuated the dextran sodium sulfate (DSS)-induced IBD symptoms in mice, probably by increasing *Lactobacillus* and decreasing *Clostridium perfringens* and *Escherichia coli* (19). Enrichment of *Lactobacillus* was found in these works, again highlighting the protective role of SCFA-producers in epithelium integrity and anti-inflammatory responses.

Strikingly, several studies found no significant effect of ENMs (Au, CuO, Ag and lysine-modified SWCNTs) on intestinal immunity (17, 20, 21, 27, 29, 87). One possibility is that most ENMs may be rapidly excreted following ingestion, so few accumulate in the GIT and they are insufficient to modulate the immune responses. Indeed, 270-day consecutive dietary supplementation with ZnO ENMs (1600 mg/kg food) for mice revealed no detectable ENM distribution in the GIT (88). Hence, this work indicates that there is no general effect regarding the biodistribution and accumulation, it should be specific to each ENM. Additionally, the mucus layer that is mainly composed of highly-glycosylated secreted proteins overlying the intestinal epithelium could trap ENMs and minimize their contact with gut microbes and mucosal cells (8). This can explain why the modulatory effect of ENMs on gut microbes *in vitro* is always greater than that *in vivo*. When the earthworms were exposed to soil with CuO or Ag ENMs, the gut microbiota remained largely resilient, whereas both ENMs significantly changed the soil bacterial community composition (89). Moreover, though some ENMs can modify the gut microbiota, members of the core commensal consortium are not affected; or the roles of redundant symbionts affected by ENMs could be compensated by other unchanged commensals. For example, exposure of earthworms to soil supplemented with CuO ENMs (160 mg/kg) induced substantial changes in the gut microbiota with a significant decrease in the symbiont *Candidatus Lumbricincola*, but it had no effect on the immune competence (29). Thereby, the gut microbiota might adapt itself in a way (which needs to be demonstrated) that ensures maintaining a proper immune homeostasis.

## Conclusions and Perspectives

To summarize, increasing observations have claimed a link between GIT exposure to ENMs, gut microbiota dysbiosis and intestinal inflammation (**Figure 2**). Such effects of ENMs are often dose-dependent. We acknowledge that in a few cases ENMs could induce microbiota dysbiosis characterized by a decrease in commensals (*Lactobacillus, Bacteroides*, *Bifidobacterium*, *etc.*) and/or an enrichment of other members (*E. coli, Shigella, Listeria, etc.*), which in turn cause an intestinal inflammation, compromise epithelium integrity and induce IBD-like symptoms (**Figure 2**). But these works suffer shortcomings and are not relevant for human exposure doses or uptake conditions. By contrast, little or no overt effect on intestinal immunity has been found in a large number of *in vivo* assays, where ENMs are orally administered in a more realistic dose or fashion. Notably, most *in vivo* studies investigate the immunotoxicity of ENMs in healthy individuals, while it might be more prominent in those with intestinal inflammation (such as IBD). Indeed, inflammatory symptoms like mucus defects (90), dysfunctional macrophages (91), *etc.* could increase and extend the exposure of intestinal epithelium to ENMs. Interestingly, the DSS-induced IBD symptoms in mice can be either exacerbated (92) or attenuated (19) following oral intake of ENMs, suggesting that ENM exposure do not necessarily have detrimental consequences, even for those with inflamed intestine. Future works should cover more types of ENMs, simulate the real-life ENM exposure situation, exploit both healthy and inflammatory host model, and draw cautious conclusions.

The *in vivo* studies on different animal models show extensive variation regarding the ENM effects on gut microbiota or intestinal immunity (**Table 1**). This may be due to discrepancies in the overall experimental settings (animal species, age, EMN exposure time, dose and uptake manner), the ENM physicochemical nature (size, shape, surface decoration and charge), the possible *in vivo* bio-transformation of ENMs and the methodology for gut microbiota analysis (**Table 1**). A unifying exposure model is required.

However, pitfalls of current animal models should be considered when translating gut microbiota research results to humans. The murine gut microbiota resembles the human one at phylum level, but differs at genus and species level (93). The anatomy and physiological functions of several GIT segments in the mouse are also different from those of humans (93). Therefore, a future perspective is to establish human models, necessarily *in vitro*, based on primary cells. To this end, microfluidic intestine-on-chips that can establish a prolonged coculture of human intestinal epithelium and gut microbes could be a promising *in vitro* human model to evaluate the ENM immunotoxicity (94, 95). When supplemented with immune cells, the intestine-on-a-chip could enable us to monitor the dynamics of ENM behavior in the gut tissue, gut microbiota changes, intestinal barrier function, immune cell activation and inflammation, thus providing predictive values on the ENM immunotoxicity. An additional but important point is the variability of gut microbiota, not only inter-individually but also at the intra-individual level (for instance in different health conditions). This calls for the need of a personalized profiling of the ENM effects on gut immunity, as it will depend on the individual microbiota in a given moment. Future immuno-nanosafety models, like the intestine-on-a-chip mentioned above, will therefore need to include the individual microbiota and the innate immune cells (in particular macrophages) derived from the individual subject.

## Author Contributions

MT, JQ, and LL devised the study and wrote the manuscript. SL, LW, and ZH contributed to literature search and gave insightful suggestions in revising this work. All authors contributed to the article and approved the submitted version.

## Funding

This work was supported by the National Natural Science Foundation of China (81900071).

## Conflict of Interest

The authors declare that the research was conducted in the absence of any commercial or financial relationships that could be construed as a potential conflict of interest.

The Editor declared a shared affiliation with some of the authors MT, LW, JQ, LL at the time of review.

## Publisher’s Note

All claims expressed in this article are solely those of the authors and do not necessarily represent those of their affiliated organizations, or those of the publisher, the editors and the reviewers. Any product that may be evaluated in this article, or claim that may be made by its manufacturer, is not guaranteed or endorsed by the publisher.
